# Editorial: Defective DNA damage response–Repair axis in post-mitotic neurons in human health and neurodegenerative diseases

**DOI:** 10.3389/fncel.2022.1009760

**Published:** 2022-08-23

**Authors:** Anna Konopka, Julie D. Atkin, Joy Mitra

**Affiliations:** ^1^Centre for Motor Neuron Disease Research, Faculty of Medicine, Health and Human Science, Macquarie Medical School, Macquarie University, Sydney, NSW, Australia; ^2^La Trobe Institute for Molecular Science, La Trobe University, Melbourne, VIC, Australia; ^3^Department of Neurosurgery, Center for Neuroregeneration, Houston Methodist Research Institute, Houston, TX, United States

**Keywords:** neurons, DNA damage, DNA repair, post-mitotic, neurodegeneration

Efficient DNA repair processes are essential for the maintenance of genomic integrity and therefore cellular homeostasis. Due to their post-mitotic nature, neurons cannot be replenished like other cell types and thus they must deal with DNA lesions throughout their lifespan, implying that DNA damage is particularly deleterious to these cells. DNA damage accumulates in neurodegenerative diseases, and thus may contribute to the selective death of specific types of neurons. However, DNA lesions are required for the regulation of neuronal gene expression and synaptic function and thus DNA damage is significant in normal physiological processes. Whilst recent evidence has shed new light on the increasingly complex roles of DNA damage and its repair in neurons, these processes are not sufficiently understood.

This special issue discusses our current understanding of the role of DNA damage and repair processes in neurons in both normal cellular physiology and the development of neurodegenerative diseases such as Huntington's disease (HD), spinal muscular atrophy (SMA), amyotrophic lateral sclerosis (ALS), Parkinson's disease (PD), and Alzheimer's disease (AD).

Gangwani and Cuartas review our current knowledge of the role of R-loop-associated DNA damage in transcriptionally active genomic regions in spinal muscular atrophy (SMA). R loops are DNA-RNA hybrids that form normally during transcription, but their timely resolution is critical in avoiding R-loop-related genomic instability and maintaining genomic fidelity. In SMA and several other neurodegenerative disorders where the non-homologous end-joining (NHEJ) DNA repair pathway is defective, R-loop formation leads to aberrant genomic modifications and an altered transcriptional landscape. This process is particularly important for post-mitotic neurons, and the authors thoroughly discuss this mechanism in the context of other motor function diseases, such as ataxia telangiectasia (AT), ataxia oculomotor apraxia 2 (AOA2), ALS and AD, in this review. In conclusion, the authors suggest that defective R-loop biology could be a convergent factor among all the known neurodegenerative diseases, where the persistent accumulation of DNA double-strand breaks in repair-inefficient motor neurons ultimately leads to aggressive disease phenotypes. However, further investigation into this possibility is warranted to better understand the common disease-causing mechanisms in these conditions.

Pradhan et al. summarize the current understanding of disease mechanisms related to DNA damage in HD, focusing on the role of huntingtin (HTT) in transcription-coupled DNA repair (TCDR) in motor neurons. While wildtype HTT facilitates DNA repair by stimulating TCDR in HD, mutant HTT (containing polyglutamine repeat expansions) forms toxic aggregates, interferes with transcription, induces synaptic dysfunction, and impairs DNA repair processes. Thus, persistent DNA damage within actively transcribing genes or promoter regions may impede transcription of a variety of neuronal genes, impacting overall neuronal health and function in HD. In addition, the expansion of polyglutamine repeats in HTT causes DNA damage, thereby triggering neurotoxicity in HD. The authors state that neither the transcriptional signatures nor the activity patterns of any two classes of neurons are likely to be identical, thus different classes of neurons may display different patterns of DNA damage.

Similar views as those described by Gangwani and Cuartas, and Pradhan et al., are also presented by Konopka and Atkin and Li et al.. These articles link DNA damage and defective DNA repair with the selective vulnerability of specific classes of neurons in neurodegenerative diseases.

Konopka and Atkin discuss the importance of the physiological roles of DNA damage and repair in neural and synaptic plasticity, which underlies processes including learning and memory, brain development, sensorial training, and recovery from brain lesions. Putatively, DNA damage and repair processes alter neurotransmission, as well as neural and synaptic connections, thereby modulating the functions of specific types of neurons. Furthermore, activity-induced DNA lesions in neurons are known to regulate the expression of early response genes, including those involved in neural and synaptic plasticity. Thus, dysfunction of these physiological DNA damage and repair processes may lead to the selective death of neurons in neurodegeneration (depending on the genes targeted), and the functions served by specific neurons. The authors also summarize our current knowledge of the relationship between neuronal plasticity and DNA damage in neurodegenerative diseases such as ALS, AD, HD, and PD.

Li et al. provide a comprehensive discussion of the role of DNA and RNA polymerases in cell type-specific DNA repair and its significance in neurological and neurodegenerative diseases. Importantly, terminally differentiated neurons remain in the G0/G1 phase of the cell cycle, hence, transcription-uncoupled repair pathways are major contributors to genome maintenance. The authors focus on the sources of DNA damage, DNA repair pathways, and structure-function relationships of a wide variety of DNA/RNA polymerases that are specifically activated in neurons. They also discuss the implications of cell-type specific activation of DNA repair pathways in response to both endogenous and exogenous genomic insults to neurons. A better understanding of the role of polymerases in DNA repair may reveal how long-lived, post-mitotic neurons cope with DNA lesions. Furthermore, this may increase our knowledge of how dysfunction in these processes contributes to disease onset, despite the diversity of neurodegenerative diseases.

In summary, dysfunction in DNA repair is common across neurodegenerative diseases. Therefore, understanding the precise mechanisms responsible for the impartment of DNA repair in neurodegeneration offers the opportunity to develop new, more effective therapies. Interestingly, neurodegenerative diseases are characterized by the selective death of a specific group of neurons, and this may originate during the impairment of DNA repair processes that govern normal neuronal functions. It is therefore worth noting that potential therapies based on DNA repair in neurodegenerative diseases should be considered carefully so that they do not interfere with the normal protective functions of DNA repair in neurons.

## Author contributions

AK wrote the first draft of the manuscript, which was developed with the input of JA and JM. All authors listed have made a substantial, direct, and intellectual contribution to the work and approved it for publication.

## Conflict of interest

The authors declare that the research was conducted in the absence of any commercial or financial relationships that could be construed as a potential conflict of interest.

## Publisher's note

All claims expressed in this article are solely those of the authors and do not necessarily represent those of their affiliated organizations, or those of the publisher, the editors and the reviewers. Any product that may be evaluated in this article, or claim that may be made by its manufacturer, is not guaranteed or endorsed by the publisher.

